# Spotlight on research integrity: international insights on strengthening research culture in the forensic sciences and beyond

**DOI:** 10.1080/20961790.2021.2015114

**Published:** 2022-01-28

**Authors:** Zoë Hammatt

**Affiliations:** University of Hawaii and Z Consulting, Honolulu, Hawaii, USA

The Singapore Statement on Research Integrity, articulated during the 2nd World Conference on Research Integrity in 2010, encompasses principles that apply to all research endeavours, including the various disciplines that comprise the field of forensic science Singapore Statement on Research Integrity (2010) (https://wcrif.org/guidance/singapore-statement). Cases of research misconduct and breaches of research integrity in any field undermine the trustworthiness of research and make it difficult if not impossible for others to rely upon and replicate research results. Moreover, problems in the research environment, whether in the academic, public, or private sector, can include bullying and harassment, discrimination, abuse of power and corruption, as well as competitive pressures related to employment and status.

Multiple actors play a role. Individual researchers, the institutions in which they work, professional societies, research funders, government authorities and oversight committees, journals and publishers, and the news media, all operate in distinct yet interrelated settings and contribute to the culture in which research is designed, conducted, and disseminated. Vigilant attention to matters of research integrity and the culture in which researchers function as they produce new knowledge can be even more critical during times of crisis such as a global pandemic. As political leaders, the media, and members of the public turn to scientists for vital information, existing pressures are compounded by circumstances such as “speed science” that pose new challenges to integrity in research [1].

In this special issue, experts in forensic science and other disciplines shed light on research integrity, ranging from observations on research culture to insights on specific skills such as acknowledgment of cognitive bias, responsible authorship, and exemplary training and mentoring.

From the perspective of a major research funder, Downey and Veitch [2] emphasise the fact that the immediate environment strongly influences researchers’ behaviour, stressing that conversations on research integrity must extend beyond definitions of research misconduct. Such definitions typically include fabrication, falsification and plagiarism, and a range of other detrimental research practices such as cherry-picking, misrepresentation, misuse of research funds, or neglectful supervision (e.g. [3]). Their call to action encourages broader discussion as we seek to envisage and create a research culture that addresses pressures and incentives and is founded upon principles and values [2]. Taking this discussion a step further, Roux and Weyermann [4] suggest an emphasis on the relevance of research, ensuring that it is informed by practice and brings value to the field and to society.

Ubelaker [5] offers specific guidance around integrity in forensic anthropology, including maintaining objectivity in research and casework as well as the importance of appropriate handling of collections, mentoring, and responsible authorship. Focusing on the role science can play in serving justice, particularly in the African context, Olckers and Hammatt [6] elucidate salient responsibilities of those involved in legal proceedings, the importance of professional certification and accreditation of forensic science laboratories, and the need for honesty and transparency in all aspects of forensic science research and practice. Dinis-Olivera [7] illustrates the increasing prevalence of predatory journals and conferences, cautioning researchers to carefully scrutinise the journals in which they publish to ensure a high level of credibility, editorial expertise, and rigorous peer review.

Training in the responsible conduct of research is required by many funders and institutions around the world (e.g. [8]). Several topics are typically addressed in varying formats, such as data management, conflicts of interest, peer review, whistleblowing, and research misconduct, but no universally accepted curriculum or teaching methodology currently exists. Nevertheless, many programmes have recognized that ethical deliberation and discussion of discipline-specific standards, “gray zones”, norms, values, and virtues are essential to such training (e.g. [9]).

As members of a European consortium, Tokalić et al. [10] reveal findings from a recent study on training, noting that higher education institutions and researchers are key to fostering a more inclusive and transparent research culture. On behalf of a national research funding agency, Luepongpattana et al. [11] describe results from a survey to determine authorship issues germane to research quality. Zhang et al. [12] review levels of academic integrity awareness around the world, and Chau et al. [13] describe methods for an active learning pedagogy that targets early career researchers and reinforces acquisition of necessary skills.

Particularly in the context of natural disasters and the current public health crisis, Noel and Torres-Ruiz [14] highlight resilience and empathy as fundamental to nurturing a positive research environment during times of duress, while Morens and Hammatt [15] distinguish between public health policy based upon reliable scientific results versus fake news, political agendas, conspiracy theories and social media campaigns.

Finally, Dejo-Vásquez et al. [16] illustrate the value of the World Conferences on Research Integrity as a multidisciplinary forum for establishing consensus, guidelines, and a research agenda applicable to all disciplines, including forensic science. Further exploration of these and other topics inherent to creating a thriving research culture will take place during the 7th World Conference on Research Integrity in May 2022 (https://wcri2022.org). The conference theme, “Fostering Integrity in an Unequal World”, has taken on even more urgency considering inequities brought into sharp focus as the COVID-19 pandemic continues around the world.


Zoë Hammatt
*University of Hawaii and Z Consulting, Honolulu, Hawaii, USA*

zhhconsulting@gmail.com


